# The importance of clinically relevant background therapy in cardioprotective studies

**DOI:** 10.1007/s00395-020-00830-y

**Published:** 2020-11-13

**Authors:** Zhenhe He, Sean M. Davidson, Derek M. Yellon

**Affiliations:** The Hatter Cardiovascular Institute, University College London, 67 Chenies Mews, London, WC1E 6HX UK

**Keywords:** Ischaemia, Reperfusion, Remote conditioning, Cardioprotection, Rats, Emricasan, Multi-target

## Abstract

Treatment of acute myocardial infarct patients (AMI) includes rapid restoration of coronary blood flow and pharmacological therapy aimed to prevent pain and maintain vessel patency. Many interventions have been investigated to offer additional protection. One such intervention is remote ischaemic conditioning (RIC) involving short-episodes of ischaemia of the arm with a blood pressure cuff, followed by reperfusion to protect the heart organs from subsequent severe ischaemia. However, the recent CONDI2-ERIC-PPCI multicentre study of RIC in STEMI showed no benefit in clinical outcome in low risk patients. It could also be argued that these patients were already in a partially protected state, highlighting the disconnect between animal- and clinical-based outcome studies. To improve potential translatability, we developed an animal model using pharmacological agents similar to those given to patients presenting with an AMI, prior to PPCI. Rats underwent MI on a combined background of an opioid agonist, heparin and a platelet-inhibitor thereby allowing us to assess whether additional cardioprotective strategies had any effect over and above this “cocktail”. We demonstrated that the “background drugs” were protective in their own right, reducing MI from 57.5 ± 3.7% to 37.3 ± 2.9% (*n* = 11, *p* < 0.001). On this background of drugs, RIC did not add any further protection (38.0 ± 3.4%). However, using a caspase inhibitor, which acts via a different mechanistic pathway to RIC, we were able to demonstrate additional protection (20.6 ± 3.3%). This concept provides initial evidence to develop models which can be used to evaluate future animal-to-clinical translation in cardioprotective studies.

## Introduction

Coronary heart disease is the leading cause of death world-wide. The main treatment is restoration of coronary blood flow using either thrombolysis or primary percutaneous coronary intervention (PPCI). However, returning coronary flow can, paradoxically, cause additional injury, known as ischaemia–reperfusion injury [19, 22]. Importantly, over the past few decades, we have witnessed a huge improvement in patient care, leading to a significant reduction in ischaemia–reperfusion injury and in the overall mortality of patients presenting with an acute myocardial infarction (MI) [41]. This improvement has been largely due to rapid intervention and ongoing therapy [41]. With regard to the latter, on hospital presentation, the majority of patients receive morphine, heparin and a P2Y_12_ inhibitor, each of which have been shown individually in preclinical studies to activate cardioprotective/pro-survival pathways [2, 7, 14, 17, 42].

One intervention that has been shown to be beneficial in preclinical studies as well as “proof of concept” studies in the clinical setting, is the use of remote ischaemic conditioning (RIC) [6, 19, 23]. RIC involves short, sub-lethal episodes of ischaemia in an organ or limb, followed by reperfusion, which can protect remote organs from the consequences of subsequent severe ischaemia [4, 19]. However, in the recent CONDI2-ERIC-PPCI multicentre outcome study [18], which investigated the phenomenon of RIC in the setting of STEMI, no benefit was observed in clinical outcome one year following RIC, which further highlights the disconnect between animal- and clinical-based studies [37]. Importantly, in the CONDI2-ERIC-PPCI study, cardiac death in patients in the control group was only 2.7%, indicating that patients entering the study were very low risk and that RIC was not able to offer any additional protection [25] Notwithstanding this, it is important to mention that in contrast to the ERIC-PPCI study, Gaspar et al. [13], in a single-centred study with limited sample size (258 patients per group), showed no reduction in myocardial infarct size (based on 48 h troponin I AUC). They did, however, find improved clinical outcomes with RIC in terms of fewer cardiac deaths or hospitalizations for heart failure. Likewise, Stiermaier et al. [40], demonstrated cardioprotection by combined intra-hospital RIC and postconditioning, in addition to primary PCI, significantly reduced the rate of major adverse cardiac events and new congestive heart failure after STEMI. However, this study was potentially limited by its post hoc nature and relatively small sample size study of 696 STEMI patients divided into three groups, which, as stated by the authors, had limited statistical power.

What is appreciated, however, is that the target for limiting the size of an evolving myocardial infarction has decreased significantly over the past few decades [37, 41]. Consequently, we believe that to pursue this diminishing, yet still important target, we need to either (i) optimise and/or enhance our existing pro-survival pathways and/or (ii) identify new independent mechanistic approaches [16]. Therefore, successful translation of cardioprotection against ischaemia–reperfusion injury may require the development of a multi-targeted approach [10, 30, 37]. In this regard, interesting data from Downey’s group clearly demonstrate in animal models that clinical-grade drugs, given at reperfusion, can provide additional and sustained infarct size reduction when added to anti-platelet therapy provided by P2Y_12_ inhibitors [1].

Based on the above, we felt it important to develop appropriate animal models that can help narrow the divide between animal-based and patient-based studies [9, 33]. Thus, to improve the translatability of animal-to-clinical studies, we developed a small-animal model which attempts to narrow this divide. This consisted of an open-chest rat model, in which rats underwent myocardial ischaemia-reperfusion on a background of the three agents routinely used when patients present with an acute MI i.e., an opioid agonist, heparin and an anti-platelet (P2Y_12_) inhibitor. We also investigated the effects of the addition of RIC and a caspase inhibitor, on this background cocktail, to investigate whether further protection could be observed.

## Materials and methods

### Animals and chemicals

Male Sprague–Dawley rats weighing 250–300 bred in the UCL biological central unit were used throughout. Animals received humane care in accordance with the United Kingdom Home Office Guide on the Operation of Animal (Scientific Procedures) Act 1986, Project Licence PPL70/8556. Both ticagrelor (antagonist of the P2Y_12_ receptor) and emricasan (caspase inhibitor) were purchased from MedKoo Biosciences (USA). Enkephalin and other chemicals were obtained from Sigma Aldrich (UK). Ticagrelor, emricasan and wortmannin were dissolved in 100% DMSO and other drugs were in saline.

### Surgery for LAD coronary artery occlusion

After anesthetization with sodium pentobarbital at a dose of 100 mg/kg via intraperitoneal (i.p.) injection, the rats were intubated by tracheotomy and ventilated with room air using a small-animal ventilator (Harvard Apparatus, Inc., model 55-0000). The rats were subject to LAD coronary artery occlusion (see details below) according to standard methods [5]. The animals were placed on a heating pad and the rectal temperature was monitored and maintained at ~ 37 °C using a CMA 450 temperature controller. During the experiments, the ECG and heart rate were continuously recorded using PowerLab (Adinstrument, USA). The left side of the chest was opened in the intercostal space between the 3rd and 4th ribs to expose the heart and a suture was placed around the left anterior descending (LAD) coronary artery. A snare was then made to allow the occlusion and opening of the LAD for ischaemia and reperfusion, as needed. With regard to the RIC protocol, rats received three cycles of RIC by inflating a silicon vascular occluder on the left lower limb for 5 min, then deflating for 5 min (see protocol below). Rats not undergoing RIC were kept on the heating pad for an equivalent time of ~ 40 min prior to open-chest surgery.

### Experimental protocols

**Fig. 1:**
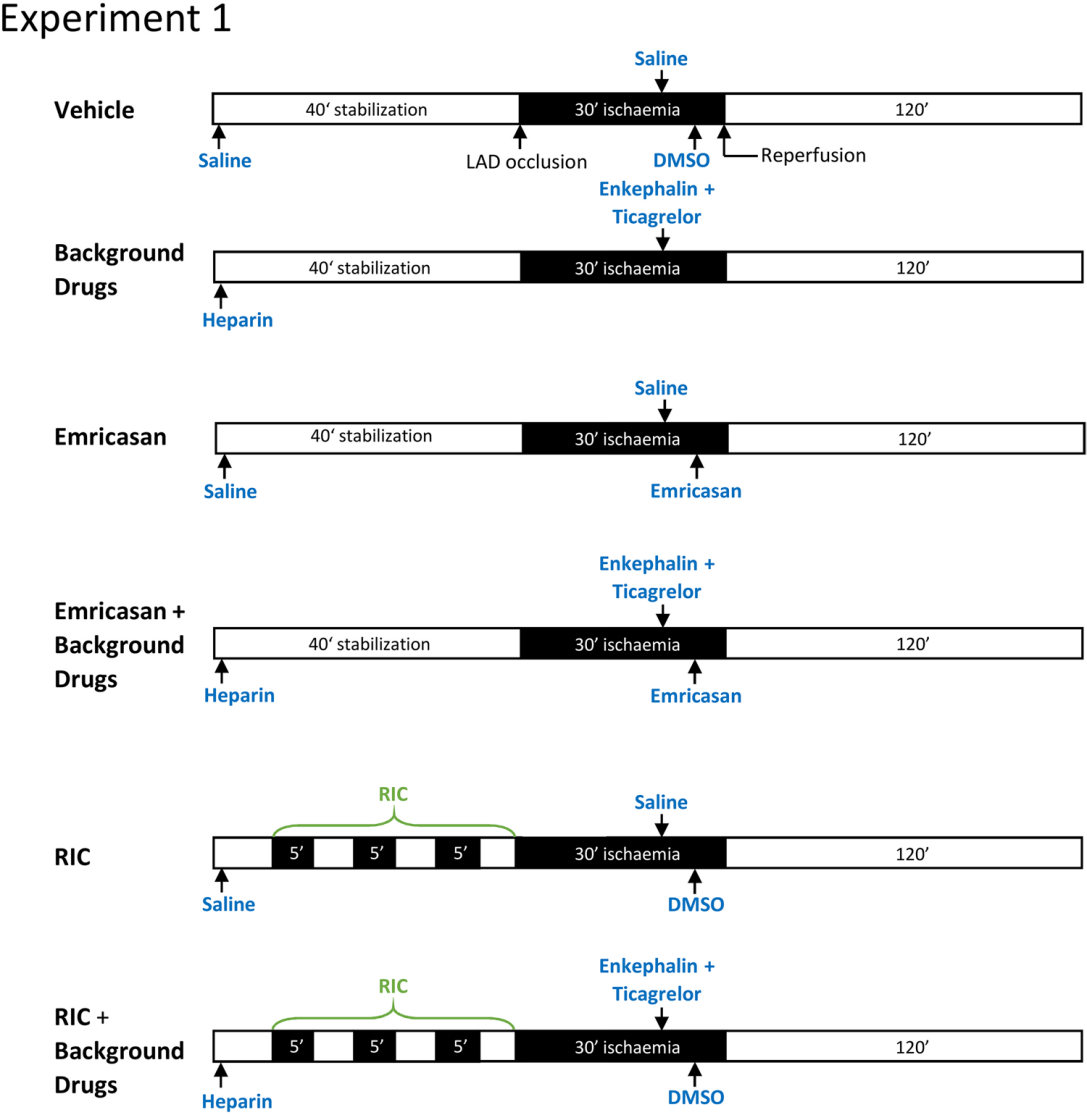
Experimental protocols for each group of rats in experiment 1. A time-line of the experimental groups indicating the time-point at which drugs were administered, RIC was administered to the rat hind limb, and the coronary artery was occluded (“ischaemia”)

By tightening the suture to occlude the LAD, the rats were subject to 30 min regional ischaemia, which was confirmed by both ST segment elevation on the ECG and the change in heart colour. At the end of ischaemia, the suture snare was loosened and 120 min of reperfusion followed. In the first experiment, the rats were randomly divided into the following 6 groups (*n* = 11 per group) (Fig. 1):

1. Vehicle control: saline and DMSO vehicles (0.3 ml/kg) were injected intraperitonealy at the equivalent time points as the drugs in all other groups.
2. Drug treated: rats in this group were treated with enkephalin (0.3 mg/kg) and ticagrelor (30 mg/kg) 10 min prior to reperfusion. In addition, heparin was administered with the sodium pentobarbital at the beginning of the experiment and prior to ischaemia.
3. Emricasan group: 5 min before reperfusion, emricasan was injected i.p at a dose of 10 mg/kg. To enable comparison with the other groups, these rats also received the same vehicles as group 1.
4. Emricasan + drugs group: these rats were treated with emricasan combined with heparin, enkephalin and ticagrelor before reperfusion;
5. RIC alone group: prior to surgery for LAD occlusion, three cycles of RIC (5 min ischaemia and 5 min reperfusion) were performed by inflating and deflating a silicon vascular occluder (OC16, from IVM, USA) on the rats’ left lower limb. In addition, all rats received the same vehicles as group 1.
6. RIC + drugs: in addition to RIC (administered as in group 5), heparin, enkephalin and ticagrelor were administered exactly as described for group 2.

**Fig. 2:**
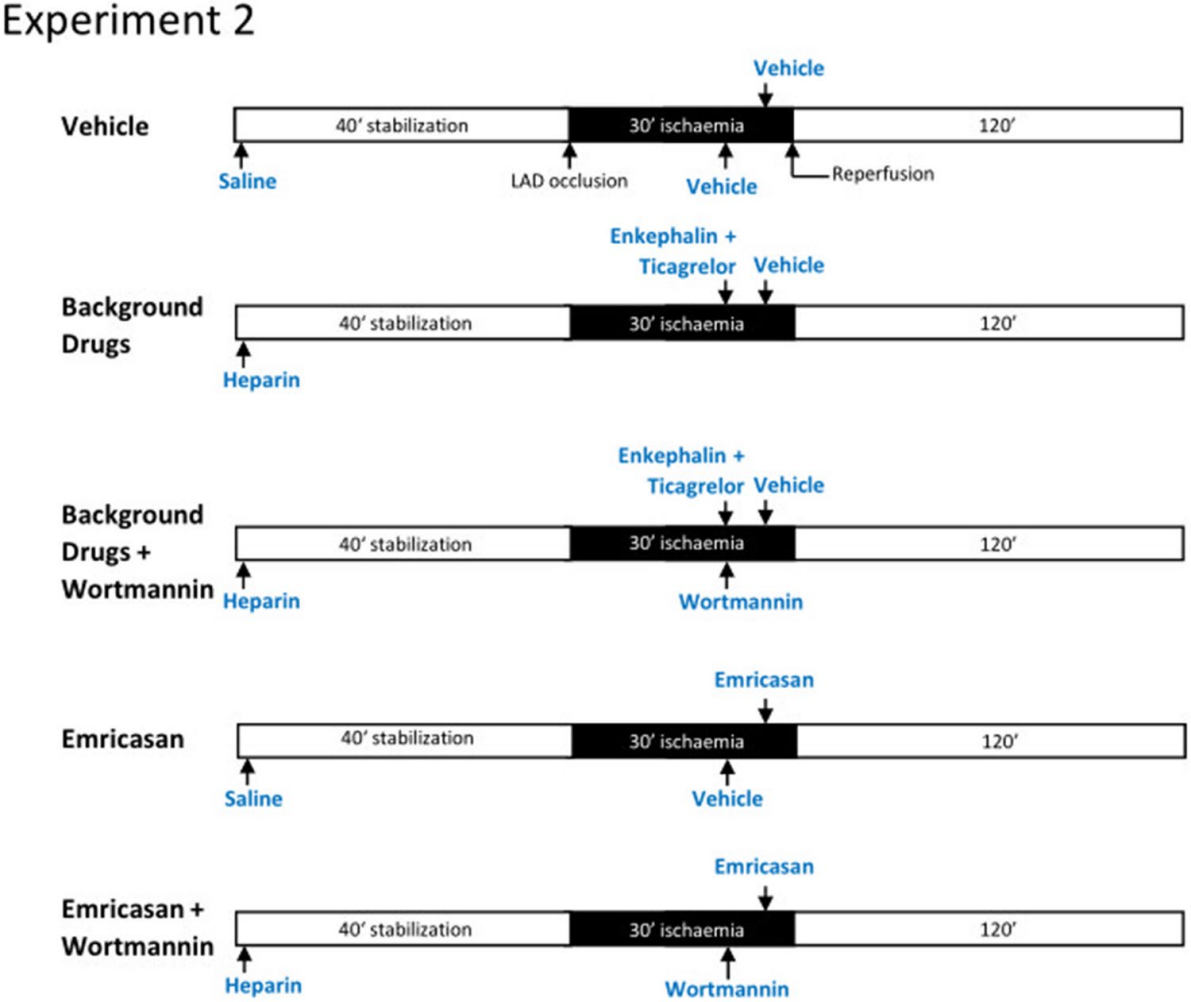
Experimental protocols for each group of rats in experiment 2 investigating the effect of wortmannin. A time-line of the experimental group indicating the time-point at which drugs were administered, and the coronary artery was occluded (“ischaemia”)

For the second group of experiments (*n* = 6–7), the groups were configured as described above with the addition of either vehicle (DMSO) or 15 μg/kg wortmannin administered 10 min prior to reperfusion (Fig. 2).

### Determination of infarct size

**Fig. 3:**
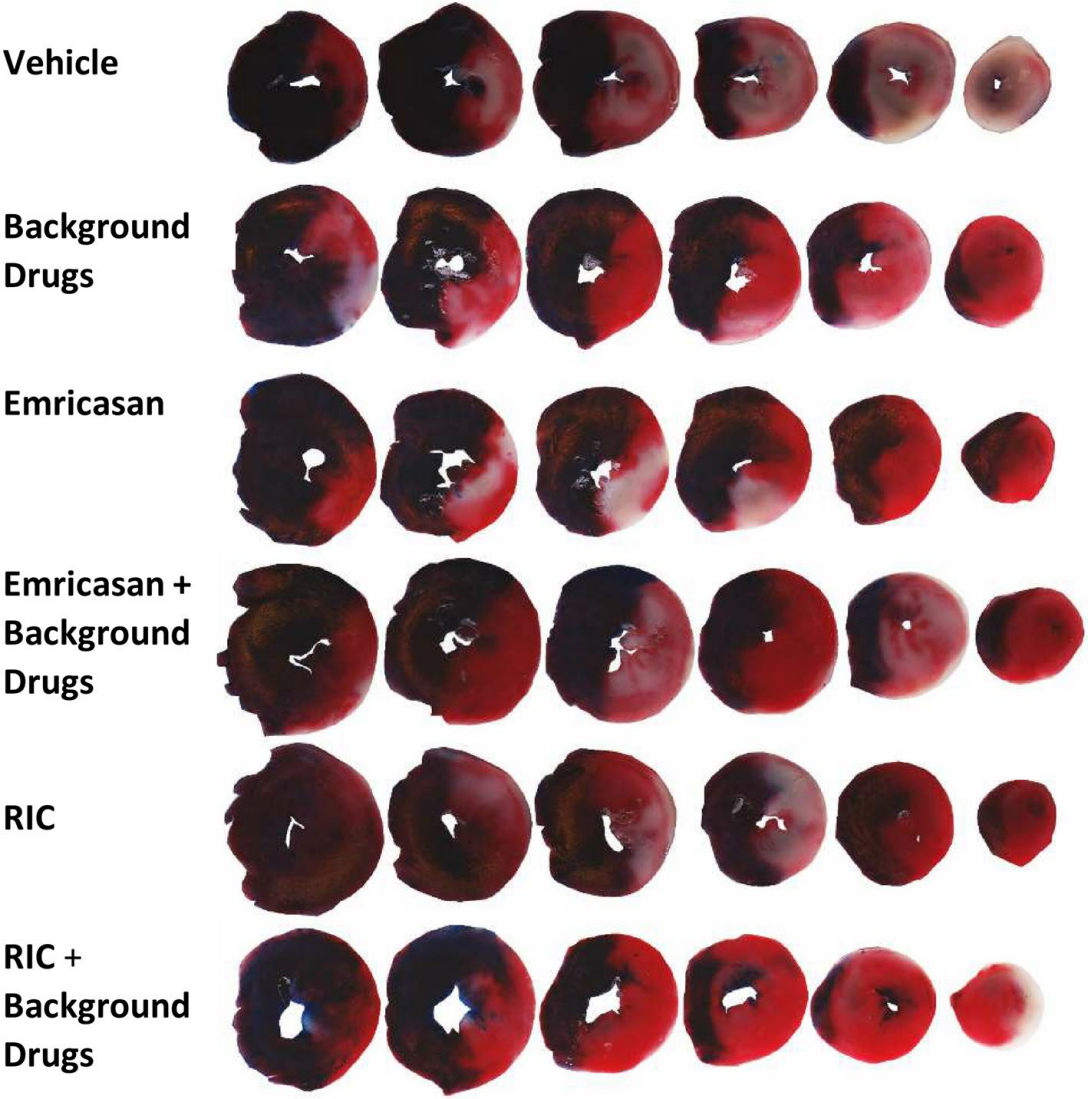
Representative serial slices of rat heart from each experimental group after staining with Evans blue and TTC. The Evans blue-stained tissue appears dark blue, representing the non-ischaemic zone. Within the ischaemic zone (“area at risk”), the red area demarcates live tissue, while the white area is dead tissue (infarct)

At the end of 120 min reperfusion, the rat hearts were excised. The aorta was cannulated and the hearts mounted on a 25 ml syringe filled with Kreb’s buffer. After washing out the blood, the LAD was re-occluded with the suture that had been left on the heart after ischaemia, and the hearts were perfused with 1% Evans blue dye to stain the non-ischaemic area of the heart. The Evans blue-stained hearts were then frozen at − 80 °C for ~ 10 min and then cut into 5–6 slices in ~ 1 mm of thickness. The heart slices were incubated in triphenyltetrazolium chloride (TTC, 10 mg/ml) solution at 37 °C, pH 7.4 for 30 min. After TTC incubation, the dead tissue was stained a white colour and the live tissue was stained a red colour. Slices were then transferred to 10% formalin solution and were fixed overnight. The heart slices without right ventricular wall were scanned using a Cannon digital scanner (Fig. 3). The entire area of the sliced, the non-ischaemic area (Evans blue-stained area) and the infarct area (white area) of the digital images were measured using Image-J software. The risk area was calculated by subtraction of the non-ischaemic area (blue area) from the whole slice area. The infarct size was expressed as a percentage of the total risk area of the left ventricle.

### Statistical analysis

Each experimental animal is indicated by a single point. The mean and standard error of the mean are indicated by the bars and handles. Statistical analysis was by one-way ANOVA followed by Tukey post test. Significance is indicated as *p < 0.05, **p < 0.01, ***p < 0.001 compared to vehicle or as indicated.

## Results

**Fig. 4:**
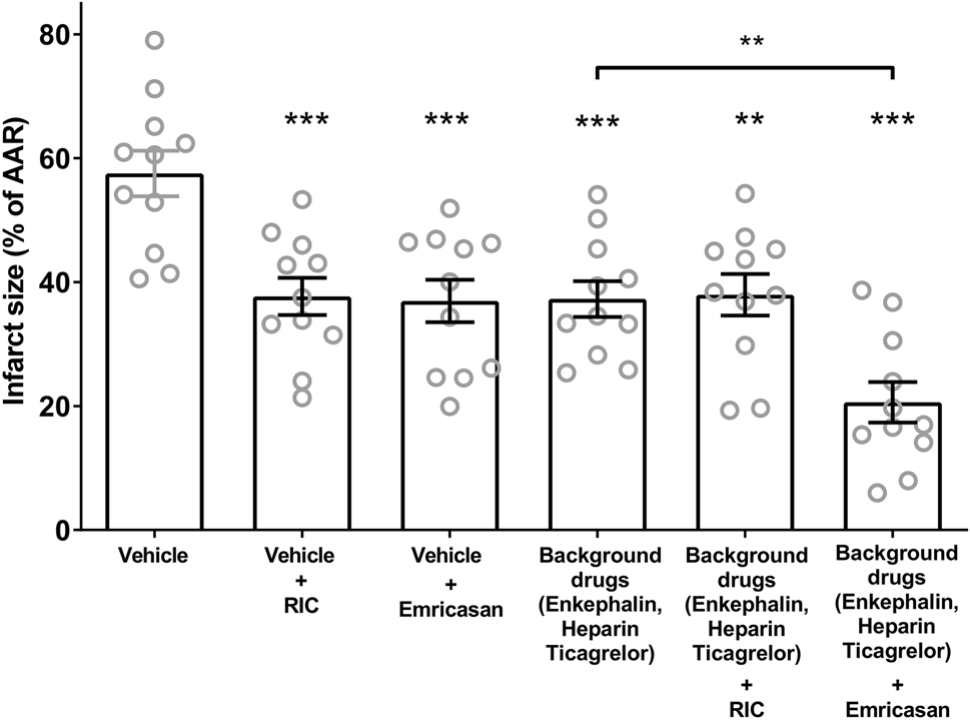
Infarct sizes expressed as a percentage of ischaemia area at risk (AAR). Individual hearts are indicated as grey circles, with the mean and standard error of the mean indicated in black. Statistical analysis was by one-way ANOVA followed by Tukey post-test. *p < 0.05, **p < 0.01, ***p < 0.001 compared to vehicle or as indicated

In the present study, we were able to demonstrate that agents routinely applied in the clinical setting to patients presenting with a STEMI, were able to protect the heart in their own right (Fig. 4). In this respect we used an opioid agonist (enkephalin 0.3 mg/kg i.p.) 10 min before reperfusion. Enkephalin was used as a substitute for morphine, which, being a protected drug, was not possible to obtain for use in our animal preparation. We also administered heparin (200 U/kg i.p.) prior to start of each experiment, in addition to an anti-platelet agent, ticagrelor (30 mg/kg i.p.) given 10 min before reperfusion. This combination of agents significantly protected the heart from ischaemia/reperfusion injury in their own right i.e., (I/R 37.3 ± 2.9% in the treated group vs 57.5 ± 3.7% in the control group; *p* < 0.001). We also investigated RIC in this model, which consisted of 3 × 5 min periods of hind-limb cuff inflation at a pressure of 150–200 mmHg interspersed with 5 min periods of deflation to zero pressure. This protocol has regularly been used in small-animal models of acute myocardial infarction [5, 6]. We were able to demonstrate that RIC alone induced significant protection compared to the control (37.7 ± 3.0% vs 57.5 ± 3.7% *p* < 0.001). Importantly, however, when we applied RIC on a pharmacological background of the three clinically relevant drugs, we were unable to demonstrate any additional protective effect (38.0 ± 3.4%). Mechanistically, this may be because both RIC and the three drugs in our cocktail have been shown to protect the heart via well-known pro-survival signalling pathways such as RISK and SAFE [38]. In view of this, we decided to investigate an alternative pharmacological approach that acts independently of the aforementioned pro-survival pathways.

We therefore proceeded to use an agent, emricasan, which predominately targets caspase-1 (in addition to other caspases) [32], and as such should therefore be expected to work independently of pro-survival signalling pathways. Indeed, emricasan was first shown by Downey’s group to protect isolated perfused mouse hearts from ischaemia and reperfusion injury [8]. We found that when emricasan (10 mg/kg) alone was administered 10 min prior to reperfusion, significant protection was observed (37.0 ± 3.4%). Importantly, when emricasan was administered on the background of the cocktail of the three clinically used drugs, an additional benefit, over and above the cocktail alone, was observed (20.6 ± 3.3% vs 37.7 ± 3.0; *p* < 0.01). In all the above studies *n* = 11 for each group (Fig. 4).

**Fig. 5:**
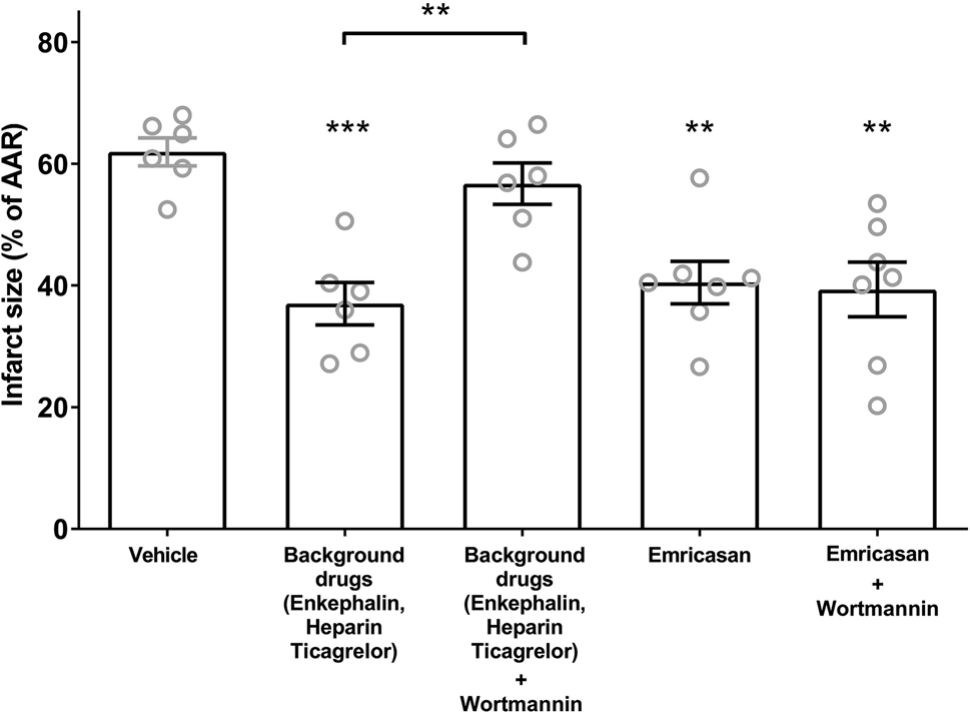
The PI3 kinase inhibitor wortmannin blocks protection by “background” drugs but not by emricasan. Infarct sizes expressed as percentage of area at risk (AAR). Individual hearts are indicated as grey circles, with the mean and standard error of the mean indicated in black. Statistical analysis was by one-way ANOVA followed by Tukey post test. **p < 0.01, ***p < 0.01 compared to vehicle or as indicated

In an attempt to ascertain the potential mechanism of protection, we undertook an additional series of experiments using the PI3-Kinase inhibitor, wortmannin. We were able to demonstrate (Fig. 5) that the protection observed with the “background clinical” drugs, was inhibited using wortmannin (37.0 ± 3.5% vs 56.7 ± 3.4% *n* = 6, *p* < 0.01) suggesting that their effect was via pro-survival kinases of the RISK pathway. However, the protection observed using the caspase inhibitor, emricasan, was not attenuated with wortmannin, (40.5 ± 3.5% vs 39.4 ± 4.5%, *n* = 7, ns) suggesting that protection by this drug occurred via a mechanisms that is independent of the RISK pathway.

## Discussion

To improve potential translatability and identify a promising multi-target approach, we developed a small-animal, rat model using pharmacological agents similar to that given to patients presenting with an acute myocardial infarction (STEMI) and undergoing primary PCI. In this open-chest rat model, rats underwent myocardial infarction on a background of three agents routinely used when patients present with an acute MI i.e., an opioid agonist, enkephalin, heparin and an anti-platelet (P2Y_12_) inhibitor ticagrelor. After induction of anaesthesia with sodium pentobarbital (100 mg/kg i.p.), and on this background trio of pharmacological agents, we then proceeded to undertake coronary artery occlusion for 30 min, by ligation of the left coronary artery, followed by reperfusion for 2 h. The heparin was given at the beginning of the experiment prior to ischaemia. The ticagrelor and the enkephalin were given 10 min prior to reperfusion. Rats were terminated upon surgical removal of the heart and myocardial infarct size measured. Using this strategy, we are able to assess the effects of known or novel cardioprotective strategies and/or drugs to ascertain whether these had any effect over and above this cocktail.

Given that the three background drugs we used have each been previously described as acting via pro-survival kinases of the RISK pathway [2, 7, 14, 17, 42], it might have been anticipated that their combination would be cardioprotective in both rats and humans. Indeed, it has previously been suggested that cardioprotective strategies should be investigated in the presence of routine background drugs [3, 20]. However, apart from the study by Audia [1] investigating the effect of P2y12 inhibitors, this is the first time that a clinically relevant model with relevant background drugs has been investigated. As we observed, RIC is no longer effective in this experimental model. This data provides one potential explanation for the failure of the RIC to demonstrate benefit in the CONDI2/ERIC-PPCI clinical trial. In a clinical setting, Kleinbongard et al. have suggested that the P2Y_12_ inhibitors show the strongest evidence that they can confound studies of cardioprotection [26]. An example of this being the combination of a statin with ischaemic postconditioning, which has been shown to overcome the resistance of diabetic mice to cardioprotection by augmenting the activation of the AKT-eNOS prosurvival signalling pathway [12]. Furthermore, this does not negate the possibility that other agents we did not test in our rat model might additionally affect the resistance of the heart to ischaemia–reperfusion injury, such as propofol, used to anaesthetize some patients [24]. It is important to note that confounding factors such as co-morbidities and co-medications can minimize RIC’s protection by two different, or even opposing, mechanisms and it is nearly impossible to differentiate between these two aspects. Protection by can be attenuated or abrogated by patients' medication and, in parallel, there is potential recruitment of protection by the medications. It may be possible that a stronger RIC stimulus would be sufficient to induce cardioprotection even in the presence of the background drugs. For example, Lieder et al. found that when RIC was applied to two limbs of a rat rather than just one, cardioprotection was greater [31].

Overall, this study demonstrates the need to produce small-animal models that more accurately reflect the clinical scenario. As discussed above, it could be argued that confounders and co-morbidities, such as age, sex and diabetes, or indeed a cohort of animals pre-treated with statins, could be further added to reflect the clinical setting more accurately. This would not have altered the main message of the study: that the cardioprotective pathways activated by background drugs must be considered in infarction experiments. A retrospective analysis of patients undergoing elective coronary bypass grafting with or without RIC prior to ischaemic cardioplegic arrest found no impact of β-blockers, statins, ACE inhibitors, ARBs or intraoperative nitroglycerin [27]. This example, and the fact the every patient has a unique profile of co-morbidities and co-medications, illustrates the impossibility of developing a “perfect” animal model that is relevant to all patients. However, all patients who present with an AMI receive a combination of drugs, as described above, which, in our model, clearly demonstrate their ability to protect the heart in their own right; this protection occurring via well-known known conditioning survival pathways. Therefore, developing pharmacological therapy or procedures, such as RIC, which act via a similar mechanism, would seem inappropriate. Instead, the focus should probably be on developing drugs that work via a mechanism independent of these survival pathways (i.e., RISK & non-RISK survival pathways). Another survival pathway, not investigated in this study, was the survivor activating factor enhancement pathway or SAFE pathway [29]. This is believed to be triggered by the immune system with prosurvival signalling via the transcription factor signal transducer and activator of transcription 3 (STAT3), which targets the inhibition of the mitochondrial permeability transition pore [15]. In addition, there is debate about the relative importance of survival pathways such as the RISK and SAFE pathways in different species (i.e., pig and human vs rat and mouse) [21, 23, 28]. However, we have shown that in human muscle studies, the survival kinases of the RISK pathway are involved in protecting human heart muscle from hypoxia-reoxygenation [39].

In this regard, our experiments indicate that the caspase inhibitor, emricasan, can protect the heart via a mechanism that does not require pro-survival kinases of the RISK pathway, and that it can therefore act additively with the three background drugs. We chose to use emricasan as it is one of the few caspase-targeted drugs that can be used in humans, having been used in clinical trials to treat non-alcoholic steatohepatitis [34]. Although it is a broad spectrum caspase inhibitor, we believe its infarct limiting effects are likely to be mediated mostly via inhibition of caspase 1, which we and others have shown provides significant benefit via inhibition of IL-18 and IL-1β [1, 11, 35, 36]. Activated caspase 1 can activate a pyroptotic cell death pathway, contributing to infarct formation [1, 11, 36]. The canonical pathway for caspase 1 activation involves the synthesis of protein subunits of the NLRP3 inflammasome, which then stimulate auto-catalysis of caspase 1 [36, 44]. However, new protein synthesis requires hours and is not relevant to acute infarct formation. On the other hand, evidence suggests that caspase 1 can be rapidly activated in naïve hearts during the first 10 min of reperfusion [8]. A putative mechanism for this is via Ca^2+^-induced activation of calpain, a calcium-dependent protease, which releases a pool of caspase-1 from the actin cytoskeleton [43]. However, the precise mechanism of caspase 1 activation during ischemia and reperfusion has not yet been determined.

To reduce the disconnect between preclinical and clinical studies (i.e.,: the influence of co-medications and/or co-morbidities), it may be necessary to start to develop animal models that more closely resemble the pharmacological and pathophysiological background of patients presenting with an acute MI. Treating patients presenting with a STEMI and tackling ischaemia–reperfusion injury in today’s environment is challenging and must require a rethink of our approach to take account of current clinical practise and patient care.
